# Qualitatively Coherent Representation Makes Decision-Making Easier with Binary-Colored Multi-Attribute Tables: An Eye-Tracking Study

**DOI:** 10.3389/fpsyg.2017.01388

**Published:** 2017-08-17

**Authors:** Masahiro Morii, Takashi Ideno, Kazuhisa Takemura, Mitsuhiro Okada

**Affiliations:** ^1^Global Research Center for Logic and Sensibility, Keio University Tokyo, Japan; ^2^Department of Economics, Tokuyama University Yamaguchi, Japan; ^3^Department of Psychology, Waseda University Tokyo, Japan; ^4^Department of Philosophy, Keio University Tokyo, Japan

**Keywords:** decision-making, eye movement, consumer behavior, multi-attribute table, graphic representation, decision strategies

## Abstract

We aimed to identify the ways in which coloring cells affected decision-making in the context of binary-colored multi-attribute tables, using eye movement data. In our black-white attribute tables, the value of attributes was limited to two (with a certain threshold for each attribute) and each cell of the table was colored either black or white on the white background. We compared the two natural ways of systematic color assignment: “quantitatively coherent” ways and “qualitatively coherent” ways (namely, the ways in which the black-white distinction represented the quantitative amount distinction, and the ways in which the black-white distinction represented the quality distinction). The former consists of the following two types: (Type 1) “larger is black,” where the larger value-level was represented by black, and “smaller is white,” and (Type 2) “smaller is black.” The latter consisted of the following two types: (Type 3) “better is black,” and (Type 4) “worse is black.” We obtained the following two findings. [Result 1] The qualitatively coherent black-white tables (Types 3 and 4) made decision-making easier than the quantitatively coherent ones (Types 1 and 2). [Result 2] Among the two qualitatively coherent types, the “black is better” tables (Type 3) made decision making easier; in fact, the participants focused on the more important (black) cells in the case of “black is better” tables (Type 3) while they did not focus enough on the more important (white) ones in the case of the “white is better” tables (Type 4). We also examined some measures of eye movement patterns and showed that these measures supported our hypotheses. The data showed differences in the eye movement patterns between the first and second halves of each trial, which indicated the phased or combined decision strategies taken by the participants.

## Introduction

### Graphical representation and decision-making

Multi-attribute tables are used in our daily life for decision-making. Among them are spec-tables, either in the traditional printed form or in the electronic form. For example, audio-players with multiple alternatives (m) and many different attributes (n) such as price and weight may be represented by an m × n table. Decision-making processes have been studied with the multi-attribute tables. Various results on decision-making with multi-attribute tables have been accumulated since the 1970s. On the other hand, the importance of introducing graphic representation has been discussed in the literature in the context of decision-making; the underlying idea is to visualize some information through graphic representations so that it assists decision-makers. The use of graphical representation and visualization of information have had increasing significance in the recent years with our new information environments where variety and complexity of information are increasing. Former studies on graphic representations used for decision-making focused on the bar chart representations (Jarvenpaa, 1989, 1990; Arribas et al., 2014). In particular, Jarvenpaa (1989, 1990) reported the efficacy of graphic representations in multi-attribute decision-making. However, very few studies have been conducted on decision-making combining graphic representation with direct use of the multi-attribute tables, although it is extremely important. Our study was the first step to bridge these two decision-making study paradigms: the traditional study paradigm for clarifying decision-making process using multi-attribute table on the one hand and the study paradigm for clarifying the effects of graphic representation (or visualization) in decision-making on the other hand. Our purpose was to find effective ways of designing graphic multi-attribute tables to support decision-making.

One of the simplest ways to introduce a graphic factor into a multi-attribute table would be to assign colors or highlights to each of the table cells (or to some table cells). Multi-attribute tables with coloring or highlighting cells are often found in our ordinary life. Even without a table format, visualizing different attribute-values by different colors is also practiced in our ordinary life. One example may be found in the traffic light food rating system used for food labels in the UK (Food Standards Agency, 2016). In this color assignment system, green, amber, and red colors are assigned to food package labels to indicate the three different value levels of each ingredient; for instance, green color is used to indicate that the fat content is less than a certain threshold, which is considered to be healthy; whereas, red indicates that the fat content is more than a certain threshold, which is considered unhealthy; and amber indicates that it is between the two thresholds. The system is also used to indicate sugar content, salt content, and so on. This coloring system gives information about food attributes in a simple and coherent way and encourages consumers to make healthier decisions. It facilitates an intuitive understanding of the attribute information of the alternatives, which supports decision-making (Jones and Richardson, 2007; Larrivee et al., 2015). In this study, we classified the value-levels using some thresholds and assigning different colors for different value-levels in a similar way but in more basic and binary levels.

Apart from the decision-making context, graphical representations for data visualization have been studied in the areas of perception and graphical designing. For example, Chen et al. (2007) discussed how one could visualize statistical data using graphs to make them easy to understand. Various relationships between ways of graphic representations and understandability have been discussed (Tufte, 1997; Shah and Hoeffiner, 2002; Kosslyn, 2006). Kosslyn (2006) proposed principles for designing statistical graphs to make them easily understandable.

As mentioned above, bar chart representations have been used in the decision-making studies with graphic representations (Jarvenpaa, 1989, 1990). Bar chart graphics may often satisfy the compatibility principle and the salience principle in the sense suggested by Kosslyn (2006); the larger and important amount is represented by a longer bar, which is more salient. However, in the case of multi-attribute decision-making, longer bars, which are salient, could be less important. For example, assume that the values of each attribute of audio-players are represented on a bar chart. For the battery duration-time, since a longer duration is better, longer bars represent a larger qualitative (as well as quantitative) value for decision-makers. On the other hand, for the price, since a lower price is better, shorter bars represent larger qualitative values. Hence, bar charts and qualitative value are not always in harmony with the compatibility principle and the salience principle, while bar charts and quantitative values are always designed to be in harmony with the compatibility principle and the salience principle. The framework of multi-attribute tables provided us different situations; we explored the difference between quantitatively coherent representation and qualitatively coherent representation of graphic designs for multi-attribute decision-making. We conjectured that the qualitatively coherent representation helps decision-making in general, as we presumed that the decision-makers made decisions using qualitative (usually, better) attribute-values. We also conjectured that if the qualitatively better values are salient (e.g., by coloring them), such a graphic representation would be helpful for decision-makers. Evidently, bar chart graphics cannot realize such a situation as explained above.

As mentioned in the beginning of this paper, we initially studied this within the framework of colored multi-attribute tables. Then, this question emerged: what methods of graphic designing would be important to assist decision-makers within this framework? Because of the limitation of cell space of a table, there are limited ways to design representations. This is because we cannot place too much information in a small cell as too detailed information within a limited space would not be suitable for designing graphics (as the principles tell us). For example, in general, in a colored table, one cannot expect the coloring to satisfy the compatibility principle in the strict sense (there are some exceptions; the use of green color of the traffic light food rating system is compatible with the safety of the traffic system in the real world).

**Figure 1:**
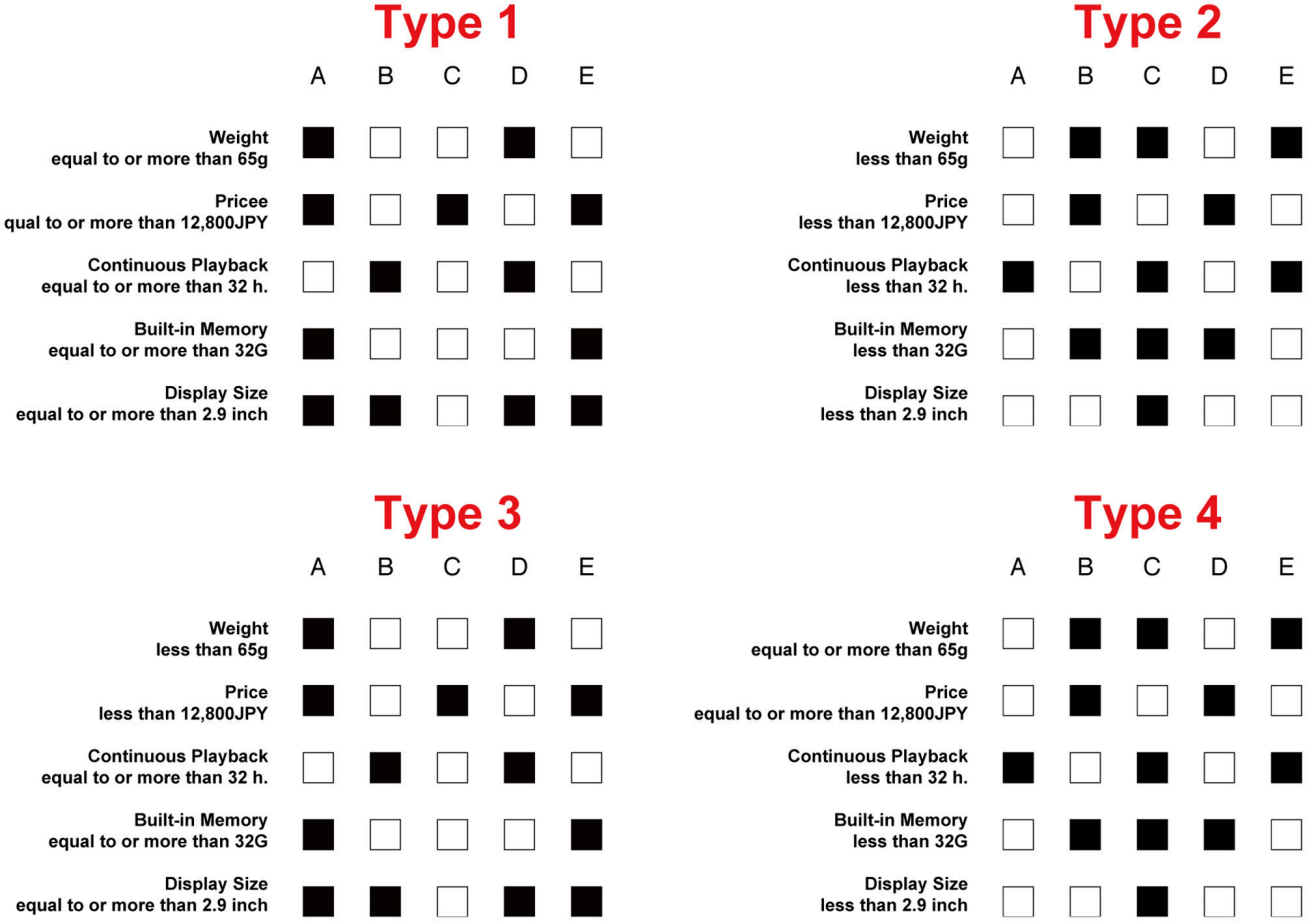
Examples of stimuli used in experiment. In Japanese, both “equal to or more than” and “less than” can be presented by two Chinese characters: “以上” for “equal to or more than” and “未満” for “less than.”

To understand which is a good way of designing colored multi-attribute tables to support decision-makers, we examined the decision-making processes using these different representations of colored tables. As the first step, we used only two colors, black and white, which were considered neutral in different cultural contexts. The tables with quantitatively coherent color assignments were called the quantitatively coherent tables, and those with qualitatively coherent color assignment were called the qualitatively coherent tables. We prepared two types of quantitatively coherent tables (Types 1 and 2) and two types of qualitatively coherent tables (Types 3 and 4), under our black-white color-assignment framework, as follows (see Figure 1 and Method for detail of our setting).

Type 1: The black cell represents a larger quantity for the attribute.
Type 2: The white cell represents a larger quantity for the attribute.
Type 3: The black cell represents a better quality for the attribute.
Type 4: The white cell represents a better quality for the attribute.

To switch the these types of tables, we switched attribute label sets as shown in Figure 1; for each attribute X and a certain value Y for the attribute X. All labels of Type 1 were of the form “(The value of attribute X) is equal to or more than Y.” All labels of Type 2 were of the form “(The value of attribute X) is less than Y.” As Type 3, for the attributes for which the larger amount is better, the labels were of the form “(The value of attribute X) is equal to or more than Y,” while the other labels were of the form “(The value of attribute X) is less than Y.” As Type 4, for the attributes for which the larger amount is better, the labels were of the form “(The value of attribute X) is less than Y,” while the other labels were of the form “(The value of attribute X) is equal to or more than Y.” When the attribute label sentence of attribute X is “true” for an alternative Z, we always colored the (X, Z)-cell black. The black-white based tables were introduced in the cognitive study by Shimojima and Katagiri (2010) on the binary-valued graphical representations using eye movement data analysis. They used the black-white cells and the symbolic letter cells (T and F), and reported, among others, that the participants had better cognitive performance with the black-white cell tables than the T-F cell tables for their cognitive tasks. We adopted their idea of the framework of black-white binary valued tables for the decision-making study rather than the cognitive study, and we used this framework for basic research on the relationship between decision-making and the color assignment patterns.

In the qualitatively coherent tables, information about “better” quality was always represented by the same color coherently (black in Type 3 and white in Type 4). We assumed that searching and comparing cells with better (or worse) values would be important for decision-making processes. This would imply that decision-making with Types 3 and 4 would be easier and especially quicker than Types 1 and 2, where searching and comparing cells with better information seemed more difficult because black may be better or worse depending on attributes. Therefore, we expected that the quantitatively coherent Types 3 and 4 helped decision-makers. Hence, we used the following working hypothesis (WH).

[WH1]

Qualitatively coherent tables (Types 3 and 4) make decision-making faster than quantitatively coherent tables (Types 1 and 2).

Previous studies have reported salience effects of the use of graphic representation on the decision-making process (Jarvenpaa, 1989, 1990; Speier, 2006; Van der Lans et al., 2008; Milosavljevic et al., 2012; Towal et al., 2013). Jarvenpaa (1990) examined the visual salience effect with alphanumeric and graphic representation by bar charts in multi-attribute tables. Information acquired was consistent with the visual salience of the attribute in the graphically represented conditions while information acquired was consistent with the importance of weights of the attributes in the alphanumeric conditions. Thus, the patterns of representations in tables affected the selection of decision-making process and final decisions even when the alternatives' values were identical. Jarvenpaa (1989) also demonstrated the interaction between the patterns of representations in tables and the selection of decision-making strategies using the same type of bar charts. The former studies suggested that both salience and graphical representation of qualitative values were important for decision-making. However, how to combine the visual salience effect and qualitative values is still unclear.

We examined the visual salience effect using our framework of binary colored multi-attribute tables, by comparing the two ways of quantitatively coherent representations, between the way in which “better” was the salient color, and the way in which “worse” was the salient color. Here, we presumed that information of better values were important for multi-attribute decision-making. Under this presumption, since the color assignment for Type 3 made the better cells salient, we expected that Type 3 made decision-making easier than Type 4. We proposed the following working hypothesis.

[WH2]

Between the two qualitatively coherent tables (Types 3 and 4), decision-makers make decisions faster with the tables in which the salient color (black) is “better” (Type 3).

### Eye movements and decision-making

If the color assignment affected the response latency, as mentioned in [WH1] and [WH2], the decision-making process could be different with different types. In studies on multi-attribute decision-making, the decision process has been explained with the concept of decision-making strategy (or heuristics). Empirical studies have revealed various strategies that are considered to be used in decision-making processes (Payne et al., 1988, 1993; Takemura, 2014). One typical strategy is the weighted additive (WADD) strategy, where decision-makers calculate expected utilities of alternatives and then choose the alternative whose expected value is the highest. Another strategy is the elimination-by-aspects (EBA) strategy where decision-makers eliminate the alternatives whose values are below the certain cutoff value in their most important attribute (Tversky, 1972). This elimination process is repeated for the second most important attribute; the processing continues until a single alternative remains. Research using protocol or questionnaire analyses suggests that decision-makers frequently combined multiple strategies in actual decision-making situations (Sheridan et al., 1975; Bettman, 1979; Bettman and Park, 1980). Bettman (1979) suggested that the most typical combined strategy is the phased strategy in which the decision-maker first selects a few alternatives by the EBA strategy and then she/he decides one among the few with the WADD strategy by taking into account values of different attributes.

In contrast to the strategy studies above, Gigerenzer et al. (1999) and Gigerenzer and Gaissmaier (2011) have stressed the point that the decision-makers often used simple heuristics by ignoring or skipping some part of strategy processes. They suggested, among others, that the “take-the-best (TTB) heuristic” was often used. One natural way to understand this TTB heuristic with our multi-attribute table setting would be a decision-making heuristic using one single attribute.

In our study, we analyzed the decision-making processes by using eye movement data. Eye movement data in multi-attribute decision-making can reveal the information on which the decision-maker focuses (Russo and Rosen, 1975; Russo and Dosher, 1983; Russo and Leclerc, 1994; Day et al., 2006; Meißner et al., 2016). If the response latency would differ among types, as we proposed in our hypothesis, eye movement data such as the number of fixations would also differ among conditions, which could be consistent with the latency differences proposed in the hypotheses.

As visual salience has a strong relationship with eye movements, analysis of eye movement data would be helpful for examining visual salience effect on decision-making. As mentioned in [WH2], we examined the visual salience effect by comparing two types of qualitatively coherent tables (Types 3 and 4). We predicted that there would be a difference in the number of fixations on black and white cells between Types 3 and 4.

Even though our setting of graphic multi-attribute tables was simple, we assumed that participants employed combined multiple decision-making strategies, rather than a simple (single) strategy. We intend to study further the decision-making processes using the eye-tracking method. As the first step of this examination, we divided each trial into the first half and the second half at the median of each trial (from the stimulus onset to the key pressing) to see the changes in information search patterns. If the eye movement patterns were different in each half, it would suggest that participants employed different decision-making strategies in each half, which would be consistent with the combined multiple strategies found in previous studies (without using eye movement data) as mentioned above.

## Materials and methods

### Participants

Eighteen Asian students (3 males and 15 females, 20–25 years old) with normal or corrected-to-normal vision participated in the experiments. All participants provided informed consent consistent with a protocol approved by the local ethics committee of Keio University. They were individually tested and paid JPY 900 (~USD 8.90) for their participation. After the experiment, one of the male participants reported that he did not understand our instructions; we excluded his data from analysis.

### Apparatus and stimuli

The eye-tracking system EyeLink 1000 (SR Research Ltd., Ontario, Canada) recorded participants' eye movements. The stimuli were presented in the center of a 23-inch display (Mitsubishi Electric Corp., RDT234WX, Tokyo, Japan). The display was viewed from a distance of 75 cm and the head was stabilized. The display resolution was 1,920 × 1,080 pixels and the visual angles were 37.5° horizontally and 21.6° vertically.

Five digital audio player products were represented graphically with five-attribute tables on white background color. A to E were “alternatives” (horizontal axis) and the specifications (specs) were “attributes” (vertical axis). The alternatives were “weight,” “price,” “continuous playback,” “built-in memory,” and “display size.” For both “price” and “weight,” the lower value was presumed “better.” For the other three attributes, the larger value was presumed “better.” The specs of alternatives were presented in 5 × 5 tables with each cell colored black or white; black cells meant “true” and white cells meant “false” for “equal to or more than X” or “less than X” on the labels. Four types of color assignments were used. One was represented by “equal to or more than X” (Type 1), where the black cells meant “large.” The other one was represented by “less than X” (Type 2), where the black cells meant “small.” Types 1 and 2 were quantitatively coherent color assignment. The other two label sets were represented by mixing “equal to or more than X” and “less than X” so that the meaning of black cells was qualitatively coherent (Types 3 and 4). For example, in Type 3, the attribute labels for “weight” and “price” were represented as “less than X” and the other three attribute labels for “continuous playback,” “built-in memory,” and “display size” were represented with “equal to or more than X”; thus, the black cells meant “better.” Meanwhile, in Type 4, the attribute label was the reverse of Type 3, where the black cells meant “worse.” Both Types 3 and 4 were qualitatively coherent color assignment.

We arranged 30 types of multi-attribute tables. Half (15/30) of the tables were arranged by inverting the cells' colors to the other 15 tables. The number of black colored cells ranged from 10 to 15. Note that the meaning of tables in Types 1 and 2 (Types 3 and 4) were semantically the same; both labels (“equal to or more than X” or “less than X”) and cells' color (“black” or “white”) were reversed. The attribute order in label sets was fixed and each attribute was represented in Japanese. Examples of the stimulus tables are shown in Figure 1.

### Procedure

After the general information and experimental procedure were described, participants sat on a chair in front of a computer screen. The calibration procedure was completed at the start of the experiment. Every participant was exposed to four blocks of 30 trials; four types of color assignments were used in each block. The order of blocks and trials was counterbalanced across the participants. At the beginning of each block, the example of stimulus table and the experimenter instructed verbally as following (in Japanese); For each attribute, black cells mean TRUE and white cells mean FALSE for the sentence of attribute labels. The test block began if there was no question from participants.

In the test trial, after a fixation cross was presented for 1 s, five alternatives were presented in a multi-attribute table. Participants were asked to choose the most desirable alternative by pressing the key without any time pressure. If the participant made a choice by pressing the key, the next trial began with no inter-trial interval. Participants' eye movements were recorded during the experiment. In eye movement data analysis, we divided each trial into the first half and the second half at the median of each trial (from the stimulus onset to the key pressing). We analyzed how the decision-making processes were different between in the first and second halves.

## Results

### Response latency

**Figure 2:**
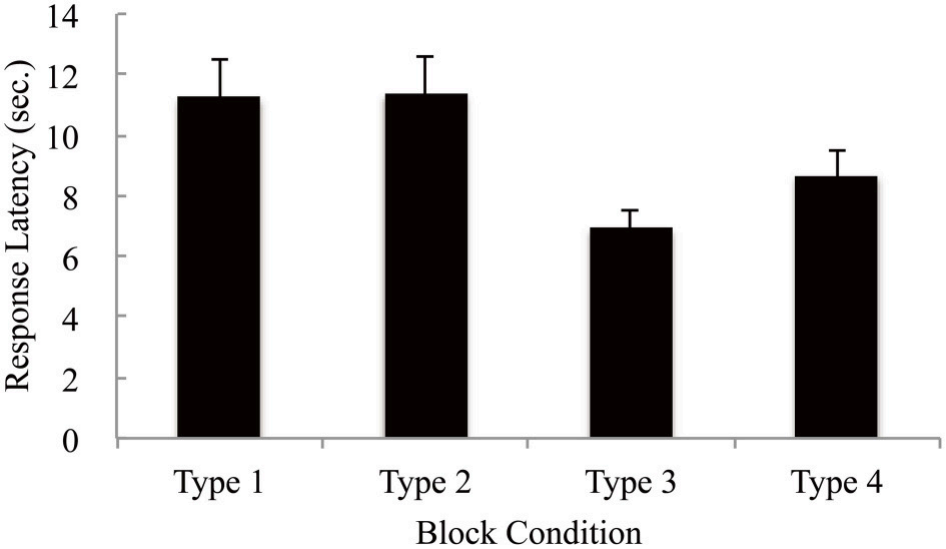
The mean response latency for each type. Error bars denote standard errors of the mean.

Response latency was defined as the time from the stimulus presentation until the key pressing. The mean response latencies were analyzed using one-factor repeated measures analyses of variance. The mean response latencies for each condition are shown in Figure 2. The main effect was significant [*F*_(3, 16)_ = 6.94, *p* < 0.001, η^2^ = 0.169]. The mean response latency for Type 3 was shorter than for Type 1 [*t*_(48)_ = 3.75, *p* < 0.01] and Type 2 [*t*_(48)_ = 3.86, *p* < 0.01]. Furthermore, the response latency for Type 4 was marginally shorter than for Type 1 [*t*_(48)_ = 2.22, *p* < 0.10] and Type 2 [*t*_(48)_ = 2.33, *p* < 0.10]. The results showed that response latencies were shorter with qualitatively coherent types (Types 3 and 4) than with quantitatively coherent types (Types 1 and 2), which was consistent with our [WH1]. Furthermore, response latency was significantly faster in Type 3 than in Type 4 [*t*_(16)_ = 2.301, *p* < 0.05], which was consistent with our [WH2].

### Choice concordance rate

**Figure 3:**
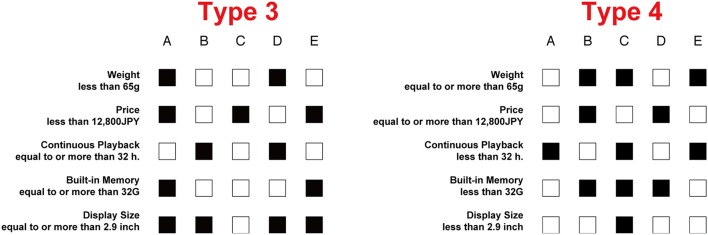
An example of a pair which have the same content.

A pair of tables from Types 1 and 2 (Types 3 and 4, respectively) was said to have the same content when the relational information of the attributes and the alternatives are the same except for a graphic setting difference, namely except that the color of each cell was reversed and each attribute labeling expression “more than or equal to” and “less than” was reversed simultaneously. Figure 3 shows an example of a pair, which has the same content from Type 3 and Type 4. A total of 30 stimuli were presented in each type where 30 pairs of tables had the same content in Types 1 and 2 (and also in Types 3 and 4). If the participant chose the same alternative which had the same content in Types 1 and 2 (as in Types 3 and 4), the choice was regarded as a concordance choice. The choice concordance rate was calculated by the rate of concordance choices out of 30 pairs.

$$\begin{array}{l} Choice\;Concordance\;Rate=\frac{The\;number\;of\;consistent\;choice}{Total\;number\;of\;pairs} \end{array}$$

The choice concordance rate between Type 1 and Type 2 was 63.1% and between Type 3 and Type 4 was 74.3%. An independent samples *t*-test of arcsine-transformed choice concordance rate indicated that the rate between Type 3 and Type 4 was higher than that between Type 1 and Type 2 [*t*_(16)_ = 2.50, *p* < 0.05, Cohen's *d* = 0.79]. This result supported [WH1].

### The number of fixations

**Figure 4:**
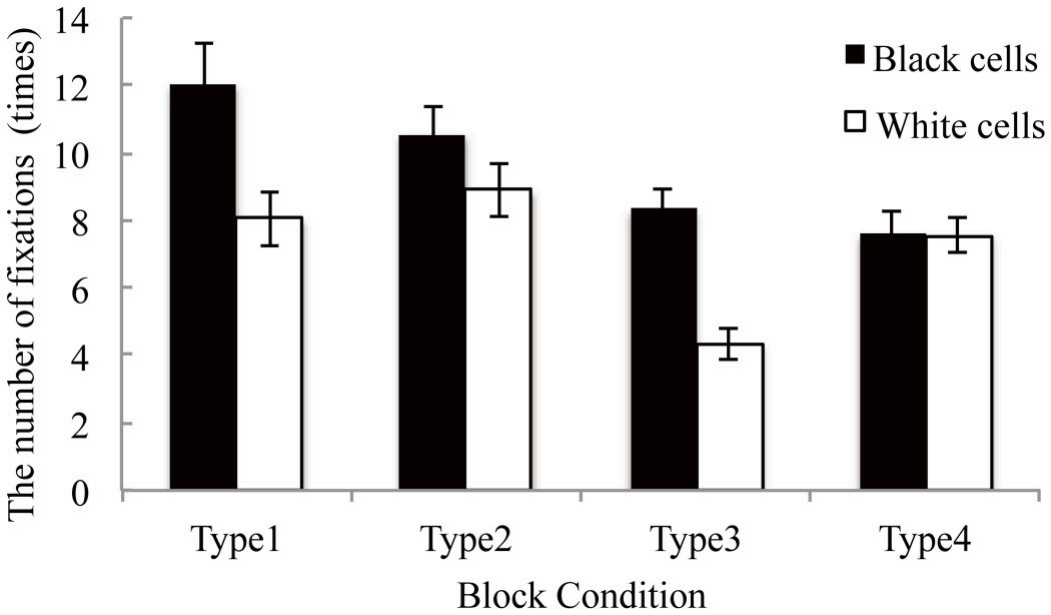
The number of fixations on black and white cells in each type. Error bars denote standard errors of the mean.

We calculated the total fixation numbers for each type in order to compare the decision- making processes across each type and also to examine effects of color assignment. In the eye movement data analysis, we used the fixations whose durations were equal to or more than 100 ms. Figure 4 shows the mean fixation count for black and white cells. The numbers of fixations were analyzed using two-factor (block types and cell colors) repeated measures analyses of variance in each half. Results showed that the main effects of block types [*F*_(3, 48)_ = 9.98, *p* < 0.001, η^2^ = 0.165] and cell colors [*F*_(1, 16)_ = 63.76, *p* < 0.001, η^2^ = 0.103] were significant. Furthermore, the interaction of the two factors was also significant [*F*_(3, 48)_ = 14.67, *p* < 0.001, η^2^ = 0.049]. Response latencies for qualitatively coherent tables (Types 3 and 4) were shorter than that for quantitatively coherent tables (Types 1 and 2), which showed the same tendency of response latency and supported our [WH1].

Multiple comparisons between Types 3 and 4 were performed. In Type 3, the number of fixations on black cells was significantly higher than that on white cells [*t*_(16)_ = 56.91, *p* < 0.001]; meanwhile, in Type 4, the numbers of fixations on black cells and white cells were not significantly different [*t*_(16)_ = 0.01, *p* = 0.909]. Although the color assignments were reversed in Types 3 and 4, the number of fixations on black cells and white cells were not reversed. These results suggested that the visual salience affected participants' fixation on cells and was in line with [WH2].

### Fixation shift patterns

**Figure 5:**
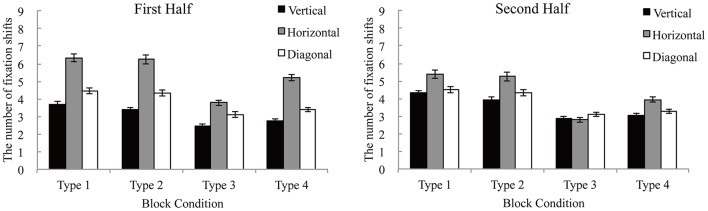
The number of fixation shifts in each type. Error bars denote standard errors of the mean.

We classified the fixation shift patterns to the vertical (*S*_*ver*_), horizontal (*S*_*hori*_), and diagonal shifts (*S*_*dia*_). *S*_*ver*_ is defined as fixation shifts within the same alternative, whereas *S*_*hori*_ is defined as fixation shifts within the same attribute. Fixation shifts across alternatives and attributes were defined as *S*_*dia*_. We counted the number of fixation shifts in each half. The fixation shift patterns were analyzed using two-factor (block types and directions) repeated measures analyses of variance in each half. The fixation shifts in each block type are shown in Figure 5.

In the first half, the main effect of direction was significant [*F*_(2, 32)_ = 47.10, *p* < 0.001, η^2^ = 0.018]. *S*_*hori*_ was higher than *S*_*ver*_ [*t*_(32)_ = 9.26, *p* < 0.001) and *S*_*dia*_ [*t*_(32)_ = 7.15, *p* < 0.001]. The main effect of block types was also significant [*F*_(3, 48)_ = 7.49, *p* < 0.001, η^2^ = 0.118]. The number of shifts was less in Type 3 than in Type 1 [*t*_(48)_ = 4.14, *p* < 0.001] and Type 2 [*t*_(48)_ = 3.73, *p* < 0.001]. The interaction of the two factors was significant [*F*_(6, 96)_ = 4.81, *p* < 0.001, η^2^ = 0.003].

In the second half, the main effect of direction was significant [*F*_(2, 32)_ = 5.53, *p* < 0.001, η^2^ = 0.031]. *S*_*hori*_ was higher than *S*_*ver*_ [*t*_(32)_ = 3.25, *p* < 0.01) and *S*_*dia*_ [*t*_(32)_ = 2.24, *p* < 0.05]. However, there was no significant difference between *S*_*ver*_ and *S*_*dia*_ [*t*_(32)_ = 1.01, *p* = 0.319]. The interaction of the two factors was significant [*F*_(6, 96)_ = 5.57, *p* < 0.001, η^2^ = 0.017]; however, the simple primary effect of direction was not significant in Type 3.

To compare the shift pattern differences more clearly, we calculated the transition score based on Payne (1976). This score was defined as following:

$$\begin{array}{l} Transition\;Score=\frac{S_{ver}-S_{hori}}{S_{ver}+S_{hori}} \end{array}$$

**Figure 6:**
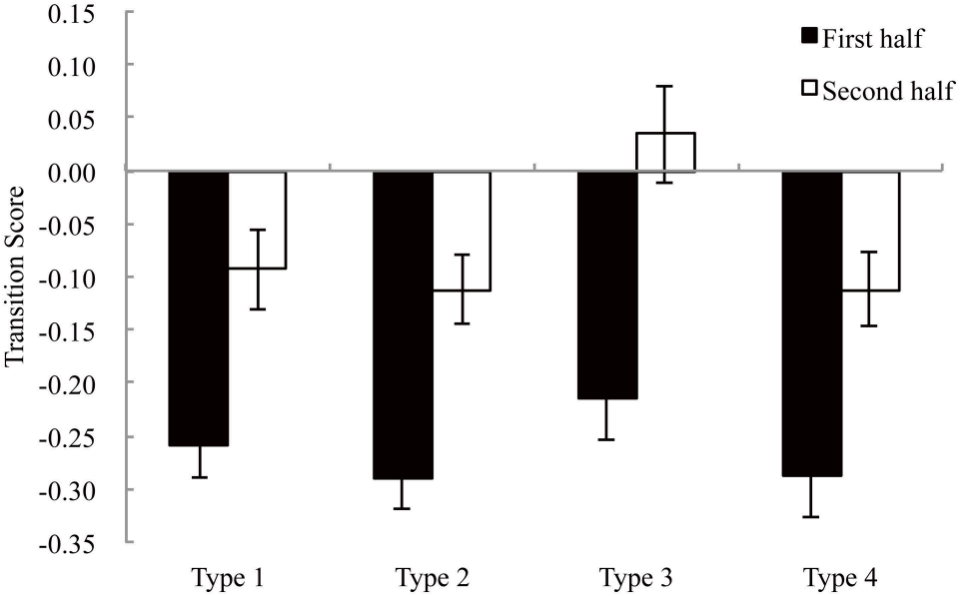
The transition score for each type. Error bars denote standard errors of the mean.

This score ranges from a value of −1.0 to +1.0. A higher value indicates relatively more alternative-based processing; a lower value indicates relatively more attribute-based processing. The transition scores were analyzed using two-factor (two-way and block types) repeated measures analyses of variance. Figure 6 shows the transition scores in each block type. The main effect of the two-halves was significant [*F*_(1, 16)_ = 81.55, *p* < 0.001, η^2^ = 0.361]. The score was higher in the second half than in the first half. In Type 3 particularly, the score was positive in the second half. The main effect of block types was also significant [*F*_(3, 48)_ = 6.47, *p* < 0.001, η^2^ = 0.062). The score in Type 3 was higher than that in other three types.

Participants' gaze frequently shifted horizontally in the first half; meanwhile the numbers of vertical and horizontal shifts were not different in the second half. These different gaze shift patterns indicated that their information search patterns were different in the first and second half as we expected.

### Process of selecting options

To analyze the process of selecting options, the variation coefficients were calculated in each condition. This score was used in Klayman (1983) and Payne (1976). This score was defined as following:

$$\begin{array}{l} V_{A}=\frac{1}{Ā}{\left[\frac{1}{n}\overset{n}{\underset{i=1}{\sum }}{\left(A_{i}-Ā\right)}^{2}\right]}^{\frac{1}{2}}\\ V_{D}=\frac{1}{\bar{D}}{\left[\frac{1}{m}\overset{m}{\underset{j=1}{\sum }}{\left(D_{j}-\bar{D}\right)}^{2}\right]}^{\frac{1}{2}} \end{array}$$

**Figure 7:**
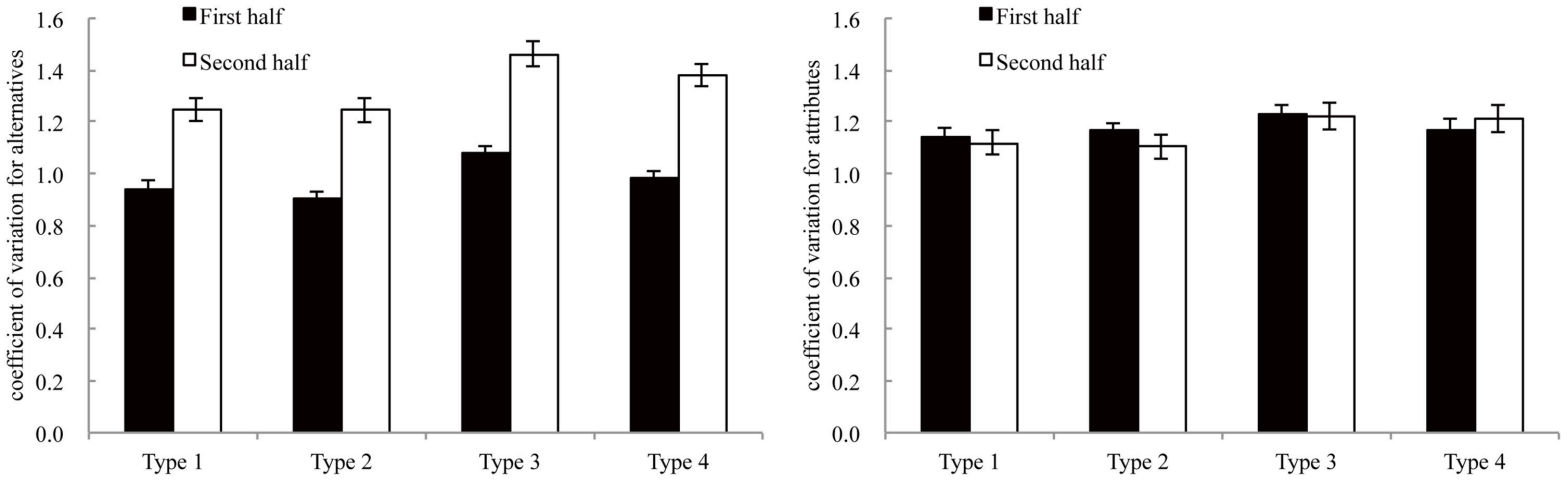
The variation coefficient for alternatives **(left)** and attributes **(right)**. Error bars denote standard errors of the mean.

Here, *n* is the number of alternatives, *A*_*i*_ is the number of fixations for alternative *i*, *m* is the number of attributes, *D*_*i*_ is the number of fixations for attribute *j*, and Ā and $\bar{D}$ are the averages of *A*_*i*_ and *D*_*j*_, respectively. The score of *V*_*A*_ (*V*_*D*_) indicates the fixation bias for certain alternatives (attributes). Higher scores meant that the participants did not consider some alternatives and gazed at only a few alternatives (or attributes); lower scores meant that the participants gazed at all alternatives equally. The variation coefficients were analyzed using two-factor (two-halves and block types) repeated measures analyses of variance. Figure 7 shows the variation coefficients for alternatives (*V*_*A*_) and attributes (*V*_*D*_).

For the variation coefficients for alternatives (*V*_*A*_), the main effect of two-halves was significant [*F*_(1, 16)_ = 117.56, *p* < 0.001, η^2^ = 0.422] and the main effect of block types was also significant [*F*_(1, 16)_ = 12.86, *p* < 0.001, η^2^ = 0.132]. However, the interaction of the two factors was not significant [*F*_(3, 48)_ = 1.09, *p* = 0.361, η^2^ = 0.005].

As the *V*_*A*_ was higher in the second half than in the first half, participants fixated all alternatives more equally in the first half and then they examined fewer alternatives in the second half. These results suggested that decision-making processes were different in the first and the second half.

For the variation coefficients for attributes (*V*_*D*_), the main effect of the two-halves was marginally significant [*F*_(1, 16)_ = 3.94, *p* < 0.10, η^2^ = 0.033] and the main effect of block types was significant [*F*_(1, 16)_ = 3.01, *p* < 0.05, η^2^ = 0.051]. The interaction of the two factors was not significant [*F*_(3, 48)_ = 1.69, *p* = 0.181, η^2^ = 0.008]. We conducted multiple comparisons for the main effect of block types and found no significant difference between each pair of types. The variation of coefficient for attributes was consistent in each type through each trial.

See Supplementary Material for aggregated data of eye movements.

## Discussion

### How the qualitatively coherent tables make it easier to make decisions

One purpose of our study was to compare the decision-making process between two types (qualitatively coherent and quantitatively coherent) of color assignment tables. In particular, we proposed the following working hypotheses concerning response latency:

[WH1] Qualitatively coherent tables (Types 3 and 4) make decision-making faster than quantitatively coherent tables (Types 1 and 2).

[WH1] was affirmatively verified, as shown in Figure 2. This suggested that the qualitatively coherent tables made the decision-makers' decision easier than the quantitatively coherent ones.

As shown in Figure 4, the numbers of fixations in qualitatively coherent tables (Types 3 and 4) are significantly smaller than in quantitatively coherent tables (Types 1 and 2). This could suggest that the decision-making processes in Types 3 and 4 were less complex than those of Types 1 and 2. This is in accordance with our affirmative result for [WH1]. We interpreted this as suggesting that the qualitatively coherent color assignments make decision-makers' decisions easier.

Our data analysis on the choice concordance rate suggested that the decision-making process of the Types 3 and 4 were similar, while those of Types 1 and 2 were less similar. This difference was consistent with the difference in latency and in the number of fixations between the qualitatively coherent tables and the quantitatively coherent tables. A higher concordance rate would support [WH1]. This would suggest that the decision-makers search for “better” cells (or “worse” cells) overall when making the decision process. We discuss this further in the next subsection regarding the visual salience effect on Type 3.

The above interpretations had an implication concerning decision-making strategies. We shall discuss this further in the following section.

### Visual salience effect in the qualitatively coherent tables

Now, we first remind the reader that in our binary-colored tables, we used black and white color assignments on the background of the table, which was white. Hence, a black cell was a salient cell in our setting. Another purpose of this study was to compare the decision-making process between two types of qualitatively coherent color assignment tables. Therefore, we proposed the following working hypothesis concerning response latency:

[WH2] Between the two qualitatively coherent tables (Types 3 and 4), decision-makers make decision faster with the tables in which the salient color (Black) is Better (Type 3).

[WH2] was verified, as shown in Figure 2, i.e., decision-making with the tables (Type 3) where the salient cells were “better” was faster than that with the tables (Type 4) where the salience cells were “worse.” Our analysis showed that the number of fixations with Type 3 was smaller than that with Type 4. In decision-making with Type 3, the number of fixations on the black cells was significantly greater than that on the white cells, while in decision-making with Type 4, there were no differences between the numbers of fixations on either type of cell.

We could interpret this difference assuming that our decision-makers made decisions by searching the information on the “better” values. Namely, with the better-is-salient tables, decision-makers can easily search for the salient “better” cells by paying much less attention to the worse-value information. On the other hand, with the worse-is-salient tables, although she/he searched the information of the non-salient “better” cells, the decision-maker also attended to the “worse” cells because of their salience to complete her/his decision, which was less efficient compared with the former case.

If the decision-makers could switch decision-making processes based on the meaning of the salient cells (“better” and “worse” cells in Types 3 and 4, respectively), then the performance (response time and the number of fixations between Types 3 Type 4) should have not been different (note that the two table types were created by only switching the colors black and white). Our asymmetric result strongly suggested that decision-making was performed mainly using the “better” cells' information and that in Type 4, where there was a conflict between the “better” information and salience, the decision-making process of Type 4 was less easy than that of Type 3. As we discussed the previous section, we conjectured that this was one of the effects of setting the qualitatively coherent color assignment. Moreover, the salient (black-is-better) color assignment would make the comparison between the attribute values (the process used, e.g., WADD strategy) easier.

As we mentioned briefly in the previous subsection, data analysis on the choice concordance rate suggested that the decision-making processes of Types 3 and 4 were relatively similar, while those of Types 1 and 2 were less similar. On the other hand, as we discussed above, the Type 3 had favorable features for easy decision-making owing to their visual salience effect. Our choice concordance rate result above suggested that although decision-makers made decisions more easily with Type 3 than with Type 4, the final decisions between Type 3 and the corresponding Type 4 seemed to be the same with a relatively high concordance. A possible interpretation of this was that the switching of black-white colors in the qualitatively coherent assignments might not change the strategies for searching the “better” cells and so the final decisions might not differ much; however, the discussion in this subsection also strongly suggested that the “white-is-better” coloring delayed decision-maker and prompted them to follow the better-cells because the black salient (worse) cells attracted gaze.

Meißner et al. (2016) analyzed their eye movement data and demonstrated that the participants tended to focus on positive aspects of the chosen alternative and on negative aspects of the alternatives not chosen. We have not analyzed our data from this view point, but our results showed that in the setting of our binary colored tables, the situation seems simpler as the positive (better) aspect or the negative (worse) aspect participants' fixations were more focused on the black-is-better (positive) aspects cells than the white-is-worse (negative) cells with Type 3; this might be interpreted that with type 3 the negative cells of the alternatives not chosen were not gazed at much. This is not in harmony with their results above. We think this could happen because of the simplicity and easiness of Type 3. In particular, the experiment results suggested that when the “better” or positive values are represented by a salient color, decision makers tend to gaze at mainly those better or positive cells for decision-making. We expect that decision-making with other types would be consistent with Meißner et al. (2016)'s result, to some extent, but we leave this issue for our future work.

### Considering decision-making processes

The previous works of Russo and Rosen (1975) and Russo and Leclerc (1994) supported that the eye movement data were reflected in the decision-making processes. In our discussion, we adopted this presumption. We used two measures, number of fixations, and fixation shift patterns. Here, we discuss the decision-making strategies by the using of our eye-movement data analysis.

Considering the context of previous research, we analyzed our eye movement data by dividing them into an earlier part and a later part to see if our data showed any evidence of a combined strategy. We divided each trial into a first half and second half at the median of each trial. Our data analysis showed that participants' gaze frequently shifted horizontally in the first half; meanwhile the numbers of vertical and horizontal shifts were not different in the second half. These different gaze shift patterns suggested that the decision-making strategies were not a single-type strategy, but more complex or combined ones as we predicted. It has been reported that decision-makers often adopt combined strategies, in which the first phase is an attribute-based strategy, typically the EBA strategy, and in the second phase the strategies take into account the values among different attributes, typically WADD strategy. We examined the transition score and the related data analysis showed that the vertical shifts relative to the horizontal increased in the second half of the trials. A possible interpretation was that in the second half of the decision-making process, the information search increased within an alternative (hence, among different attributes) relative to that within an attribute. This could suggest that some phased strategy patterns appeared in our data.

Whereas comparing values among different attributes was characteristic of the second phase of the phased decision-making strategies, selecting the candidates for alternatives was characteristic of the first phase. Selecting alternatives and selecting attributes were measured by the variation coefficients for alternatives and attributes, respectively. Our results showed that there was no difference between the first and second halves. The normal interpretation would be that participants kept searching the same attributes. There was no difference among the types of tables, in contrast to the transition score, whereas Type 3 was different from the other types. On the other hand, regarding the variation coefficients for the alternatives, the difference in scores in the second half was greater than that in the first half. This implies that a limited number of alternatives were searched in the second half. This is consistent with the characteristic of the first phase.

In summary, information search pattern was different in the first and second half. Participants did attribute-based information search and reduce the alternatives in the first half, and then they examined the remaining alternatives in more detail with horizontal fixation shifts in the second half. However, it was difficult to identify which strategies were employed in both halves. Although Bettman (1979) suggested that the typical strategy employed in the second half was the WADD strategy, there is some other possible interpretations such as a final verification or confirmation of the chosen alternative rather than calculating expected utilities of alternatives which is supposed to be carried out in the WADD strategy. Further analyses are needed about the relationship between eye-movement patterns and identification of decision-making strategies is needed for our future studies.

## Conclusion

This study analyzed the decision-making process with multi-attribute tables represented graphically by black and white cells. Using this framework, we compared the two possible systematic color assignments: qualitatively coherent assignments and qualitatively coherent assignments. Colored tables with qualitatively coherent color assignments facilitated decision-making. Furthermore, between the two qualitatively coherent color assignment tables, when the visually salient cells (black cells) were used for representing “better” qualitative values, it facilitated decision-making. With the black-is-better tables (Type 3), decision-makers could focus more on the better (black) cells while paying less attention to the worse (white) cells, whereas in the white with the white-is-better tables (Type 4), decision-makes focused on both worse (black), and better (white) cells equally.

One of the next steps of our study will be to use the framework of “highlight tables,” instead of just the black-white colored tables. Here, by a highlight table, we mean a table in which each cell has numerical values with a background color. For example, we could modify our tables of four types by inserting concrete numerical values in each cell and we could add a background color, such as light pink, instead of black. This setting of highlight table would give us a more practical setting, which may be found in our daily life. We could conjecture that with such a (practically more realistic) highlight table setting, the main parts of our findings in this paper would be confirmed. If so, the basic results of this study could be beneficial for designing practical multi-attribute tables for commerce and e-commerce.

A characteristic of the use of multi-attribute table form, in our opinion, lies in the point that the decision-maker can view the trade-off of some limited numbers of attributes, and of alternatives on a screen or on a printed paper simultaneously. This would be one of the merits of the table representation compared to other graphic representations for multi-attribute decision-making; for example, former work of Jarvenpaa (1990) took multiple-page setting of bar chart graphics because of the multiple attributes involved. The form of the multi-attribute tables is relational, which could provide much information in a single page. In particular, as we discussed above, we think that the qualitatively coherent highlighting or coloring would support decision-making more when comparing values among different attributes, such as “rational” or “normative” utility based processes (such as the WADD strategy).

In this paper, we have not considered the utility measure in our decision-making study. We will plan to conduct experiments using participants' individual expected utilities calculated by a conjoint measurement method in order to investigate the relationship between decision-making with our colored tables and the utility-based normative decision-making.

We only considered 5 × 5 tables for our experiments. Payne (1976); Olshavsky (1979), and Onken et al. (1985) suggested that the size of tables affects decision-making processes. For our future research, we plan to consider different table-sizes, especially a larger size, to see how our results, observations, and conjecture could be preserved in a larger size table. In addition, we plan to conduct further experiments to clarify how multiple or phased strategies are used in decision-making. In particular, in this study, we split each trial into the first half and the second half by the median of each trial to see some change in decision processes during a trial. We would like to obtain data using different intervals of each trial to see further details of the eye movement changes. It would be ideal if we could find a more natural point than the median to split in order to clarify the phased processes. Since the numbers of fixations in each trial were too small in the current study, it was hard to consider various different splitting points; this could be partly because the size 5 × 5 of our tables was small. Using larger size of tables in our future experiments, we believe that our study on phased processes by means of eye movement will progress.

The number of participants in this basic experiment was 18, which was relatively small. Although we believed the basic results obtained in this paper were stable, we admit that the number of participants should be increased for the future studies because we would need more size-sensitive data analysis in the next experiments.

The basic results on the colored and highlight multi-attribute tables for decision-making need to be combined with other more practical issues that we did not consider for in this study. Former work on the practical issues of graphics, such as those by Chen et al. (2007), will need to be taken into account. For example, we avoided the issue of the choice of colors in this experiment and chose black and white. For some application domains, such as that of health-sensitive food-attribute representations, “green” suggests “better-positive-safe” and “red” suggests “worse-negative-risky” as the traffic light food rating (color assignment) system in the UK. Kosslyn (2006) explained, independently of the food labeling, this characteristic of green as the “cultural” type of the “compatibility principle,” which means that green is symbolic of the green color of traffic light system in our culture (see Introduction). Chen et al. (2007) pointed out, among others, that the coloring issue was important for graphic representations, but at the same time, it is difficult; he also referred to the red color as expressing negativity. As we mentioned in Introduction, there are many application domains with no obvious coloring assignment system for multi-attribute tables following any “compatibility principle.” There are many highlight tables in our ordinary life, either in the printed form or in the electronic form, where yellow and light red are occasionally used for representing positive information. We would like to ask ourselves which choice of colors would be good for neutral or fair presentation of highlight or colored tables for decision-makers, when no obvious coloring systems exist following the compatibility principle.

## Author contributions

MM, TI, KT, and MO conceived the design of experiment. MM and TI conducted the experiments, analyzed the data, and wrote the first draft of the manuscript. All authors contributed equally to later drafts, approved the final manuscript, and are jointly responsible for the accuracy and integrity of the work.

### Conflict of interest statement

The authors declare that the research was conducted in the absence of any commercial or financial relationships that could be construed as a potential conflict of interest. The reviewer CS and handling Editor declared their shared affiliation, and the handling Editor states that the process nevertheless met the standards of a fair and objective review.
